# Rab11a is required for apical protein localisation in the intestine

**DOI:** 10.1242/bio.20148532

**Published:** 2014-12-19

**Authors:** Tomoaki Sobajima, Shin-ichiro Yoshimura, Tomohiko Iwano, Masataka Kunii, Masahiko Watanabe, Nur Atik, Sotaro Mushiake, Eiichi Morii, Yoshihisa Koyama, Eiji Miyoshi, Akihiro Harada

**Affiliations:** 1 Department of Cell Biology, Graduate School of Medicine, Osaka University, Suita, Osaka, 565-0871, Japan; 2 Department of Molecular Biochemistry and Clinical Investigation, Graduate School of Medicine, Osaka University, Suita, Osaka, 565-0871, Japan; 3 Institute for Molecular and Cellular Regulation, Gunma University, Maebashi, Gunma 371-8512, Japan; 4 Department of Anatomy, Graduate School of Medicine, Hokkaido University, Sapporo, Hokkaido, 060-8638, Japan; 5 Department of Pediatrics, Nara Hospital, Kinki University School of Medicine, Ikoma, Nara, 630-0293, Japan; 6 Department of Pathology, Graduate School of Medicine, Osaka University, Suita, Osaka, 565-0871, Japan

**Keywords:** Rab11a, Knockout mouse, Cell polarity, Brain, Intestine, Apical membrane

## Abstract

The small GTPase Rab11 plays an important role in the recycling of proteins to the plasma membrane as well as in polarised transport in epithelial cells and neurons. We generated conditional knockout mice deficient in *Rab11a*. *Rab11a*-deficient mice are embryonic lethal, and brain-specific *Rab11a* knockout mice show no overt abnormalities in brain architecture. In contrast, intestine-specific *Rab11a* knockout mice begin dying approximately 1 week after birth. Apical proteins in the intestines of knockout mice accumulate in the cytoplasm and mislocalise to the basolateral plasma membrane, whereas the localisation of basolateral proteins is unaffected. Shorter microvilli and microvillus inclusion bodies are also observed in the knockout mice. Elevation of a serum starvation marker was also observed, likely caused by the mislocalisation of apical proteins and reduced nutrient uptake. In addition, Rab8a is mislocalised in *Rab11a* knockout mice. Conversely, Rab11a is mislocalised in *Rab8a* knockout mice and in a microvillus atrophy patient, which has a mutation in the *myosin Vb* gene. Our data show an essential role for Rab11a in the localisation of apical proteins in the intestine and demonstrate functional relationships between Rab11a, Rab8a and myosin Vb *in vivo*.

## INTRODUCTION

Rab proteins are a subfamily of the Ras superfamily of small GTPases that are known to regulate specific intracellular membrane trafficking pathways. A large number of Rabs are present in a variety of organelles, suggesting that they play roles in defining the nature of these structures. The Rab family is divided into several subfamilies, one of which is the evolutionarily conserved Rab11 subfamily, which is composed of Rab11a, Rab11b, and Rab25 in mammals (Goldenring et al., 1993; Lai et al., 1994) and Ypt31p and Ypt32p in budding yeast *S. cerevisiae* (Benli et al., 1996).

A number of investigations carried out in both polarised and non-polarised cells have demonstrated that Rab11 subfamily proteins are associated with plasma membrane recycling systems, which regulate epithelial polarity and membrane trafficking into and out of the recycling endosome. In non-polarised cells, Rab11a is known to be crucial for recycling, as it has been shown to colocalise with internalised transferrin, and a GDP-bound form of Rab11a perturbs the recycling of transferrin (Ullrich et al., 1996; Ren et al., 1998). In polarised cells, such as epithelial cells, Rab11 family proteins are known to localise to the apical recycling endosome, where they play a role in apical recycling (Goldenring et al., 1996; Casanova et al., 1999; Perez Bay et al., 2013). Furthermore, Rab11 family proteins localise to subapical areas in epithelial cells of the stomach, intestine, and bladder (Goldenring et al., 1994; Goldenring et al., 1996; Khandelwal et al., 2008; Khandelwal et al., 2013). Using primary cultures and tissue cultures, Rab11 proteins were shown to be involved in the exocytosis of discoidal vesicles in bladder umbrella cells (Khandelwal et al., 2008; Khandelwal et al., 2013) and the exocytosis of H^+^K^+^-ATPase-containing vesicles in stomach parietal cells (Duman et al., 1999). Also, in *C. elegans* oocytes, Rab11 is required for the formation of caveolin-enriched secretory vesicles (Sato et al., 2008). Finally, in *Drosophila*, Rab11 is required for the secretion of rhodopsin in photoreceptor cells (Li et al., 2007).

Rab11 is known to be important for polarisation and vesicular transport in neurons, another type of polarised cell (Shirane and Nakayama, 2006; Takano et al., 2012). However because the Rab11 family includes Rab11a, Rab11b, and Rab25, it is difficult to determine which proteins are necessary for these functions in epithelial cells and neurons. To date, only *Rab25* knockout mice have been generated, which showed increased numbers of intestinal neoplasias when crossed with *APC^min/+^* mice (Nam et al., 2010). In this study, we generate brain- and intestine-specific *Rab11a* knockout mice and examine their tissues. Mice lacking Rab11a throughout their entire bodies are embryonic lethal. Brain-specific *Rab11a* knockout mice display no overt phenotypes. However, intestine-specific knockout mice show mislocalisation of apical proteins, microvillus atrophy, and microvillus inclusion bodies. Furthermore, we show that the localisation of Rab8a is altered in *Rab11a* knockout mice. Conversely, the localisation of Rab11a is altered in the intestinal epithelial cells of *Rab8a* knockout mice and in a microvillus atrophy patient, which has a mutation in the *myosin Vb* gene. These results show that Rab11a, Rab8a, and myosin Vb affect the localisation of one another, suggesting close functional relationships between these proteins.

## RESULTS

### *Rab11a* knockout mice are embryonic lethal, although brain-specific knockout mice display no overt phenotypes

**Fig. 1.**
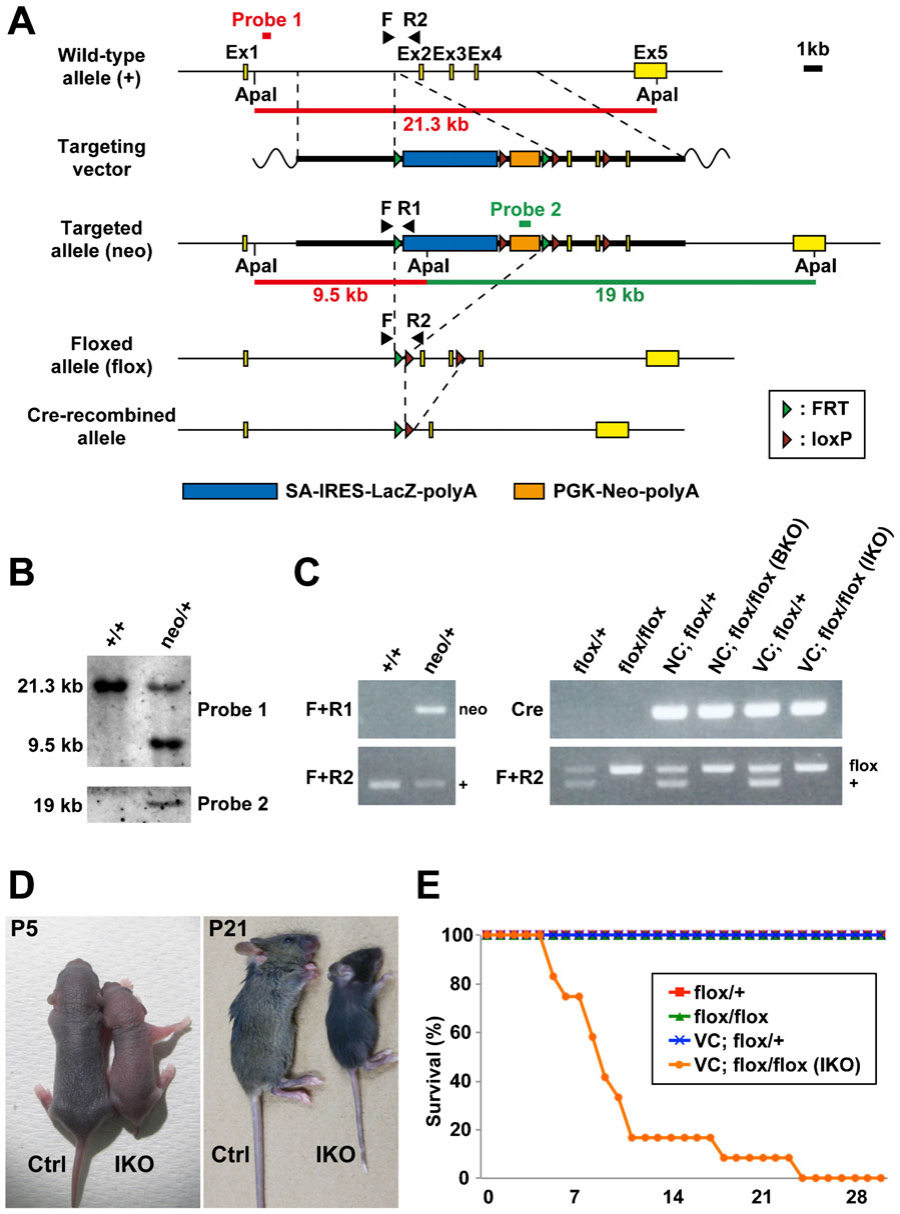
Generation and analysis of *Rab11a* knockout mice. (A) Diagram of targeting strategies. Restriction maps of the *Rab11a* wild-type allele (+), the targeting vector containing an SA-IRES-LacZ-polyA sequence (blue horizontal bar) and PGK-Neo-polyA (orange horizontal bar), and the targeted allele (neo). Brown triangle, loxP site; green triangle, FRT site. Probe 1 hybridises with a 21.3-kb Apa I fragment from the wild-type allele and a 9.5-kb fragment from the targeted allele. Probe 2, the neomycin fragment in PGK-Neo-polyA, hybridises with a 19-kb Apa I fragment from the targeted allele. SA, splice acceptor; IRES, internal ribosomal entry site; PGK, phosphoglycerate kinase promoter; Neo, neomycin resistance gene cassette. (B) Southern blotting analysis of the targeted ES cell clones. Genomic DNA from parental embryonic stem cells (+/+) and targeted clones (neo/+) were digested with Apa I for hybridisation with the probes 1 and 2 in (A). (C) Genotyping PCR analysis using genomic DNA from control and knockout mouse tails. Primers ‘F+R1’, primers ‘F+R2’, and primers ‘Cre’ were used to detect the targeted allele (neo), the wild-type (+) and floxed (flox) alleles, and *cre* gene. (D) Control (Ctrl) and intestine-specific *Rab11a* knockout (IKO) mice at postnatal days 5 (P5) and 21 (P21). The IKO mice were smaller than control littermates at both ages. (E) Survival curves for control (n = 12–14) and IKO (n = 12) mice. IKO mice began dying 5 days after birth, and all mice were dead by postnatal day 25. NC, *Nestin-Cre*; VC, *Villin-Cre*.

We generated knockout mice from ES cells (*Rab11a^tm1a(KOMP)Wtsi^*) provided by the UC Davis KOMP Repository (Fig. 1A,B). When we intercrossed heterozygous knockout mice (*Rab11a^neo/+^*) (Fig. 1C), we were unable to obtain homozygous knockout mice that completely lacked Rab11a throughout the entire body (supplementary material Table S1).

Considering that Rab11a is known to be involved in axonal elongation (Shirane et al., 2006; Takano et al., 2012), we generated brain-specific *Rab11a* knockout (BKO) mice by crossing the *Rab11a^flox/flox^* mice to *Nestin-Cre* mice (Tronche et al., 1999). The loss of Rab11a specifically in the brains of the BKO mice (*Nestin-Cre; Rab11a^flox/flox^*) was confirmed by Western blot analysis (supplementary material Fig. S1A). Contrary to our expectations, the BKO mice were born at the proper Mendelian frequency (Fig. 1C; supplementary material Table S1) and developed without overt abnormalities. Furthermore, the brains of the BKO mice appeared similar to those of control mice (supplementary material Fig. S1B). To examine the brain development, we performed Nissl staining and immunofluorescence analysis for cortical layer markers, Cux1, which is expressed in the upper layers II–IV and Tbr1, which is expressed in the layers II–IV and VI. In the BKO mice, the neocortex and hippocampus and the positioning of their Cux1- and Tbr1-positive cortical neuron did not show apparent difference from that of the control brains (supplementary material Fig. S1C,D). We further observed no obvious differences in axonal elongation (calbindin), dendritic arborisation (MAP1A or calbindin), distribution of synaptic protein (synaptophysin), and ciliogenesis (Arl13b) in the cerebrum or cerebellum (supplementary material Figs S2, S3). The results of the BKO mice indicated that Rab11a does not have critical role in the neurogenesis and the neuronal maturation.

### Intestine-specific *Rab11a* knockout mice display the intracellular accumulation of apical proteins, shortening of microvilli, and microvillus inclusion bodies

**Fig. 2.**
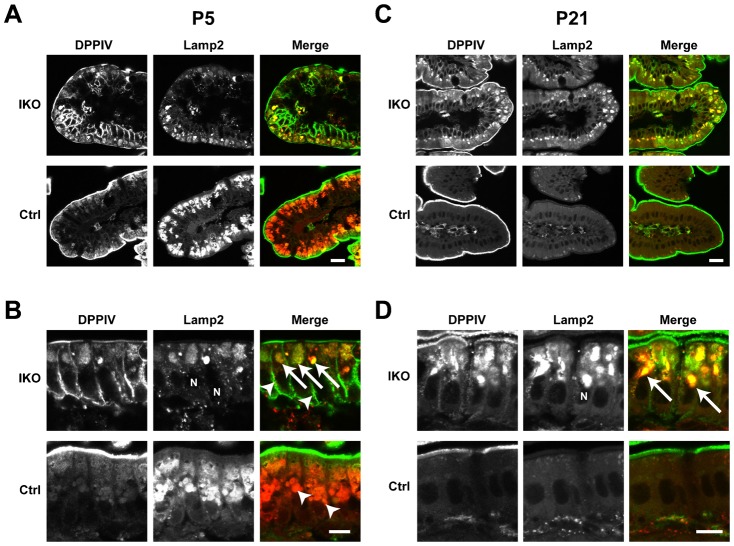
Localisation of the apical protein DPPIV in the small intestine of *Rab11a* IKO mice at P5 and P21. (A–D) Localisation of the apical protein DPPIV (green) and the lysosome marker Lamp2 (red) in control (Ctrl) and IKO mice at P5 (A,B) and P21 (C,D). Higher magnification views of the intestinal cells from Ctrl and IKO mice at P5 (B) and P21 (D). At P5 (A,B), intracellular DPPIV staining (green) is colocalised with Lamp2 (red) in IKO cells (arrows in B ‘IKO’), whereas DPPIV and Lamp2 (arrowheads in B ‘Ctrl’) did not colocalise in control cells. In addition, basolateral staining (arrowheads in B ‘IKO’) is more evident in IKO cells. At P21 (C,D), intracellular DPPIV staining is evident and the colocalisation of Lamp2 and DPPIV is greater in IKO mice (arrows in D). Scale bars: 20 µm (A,C), 10 µm (B,D).

**Fig. 3.**
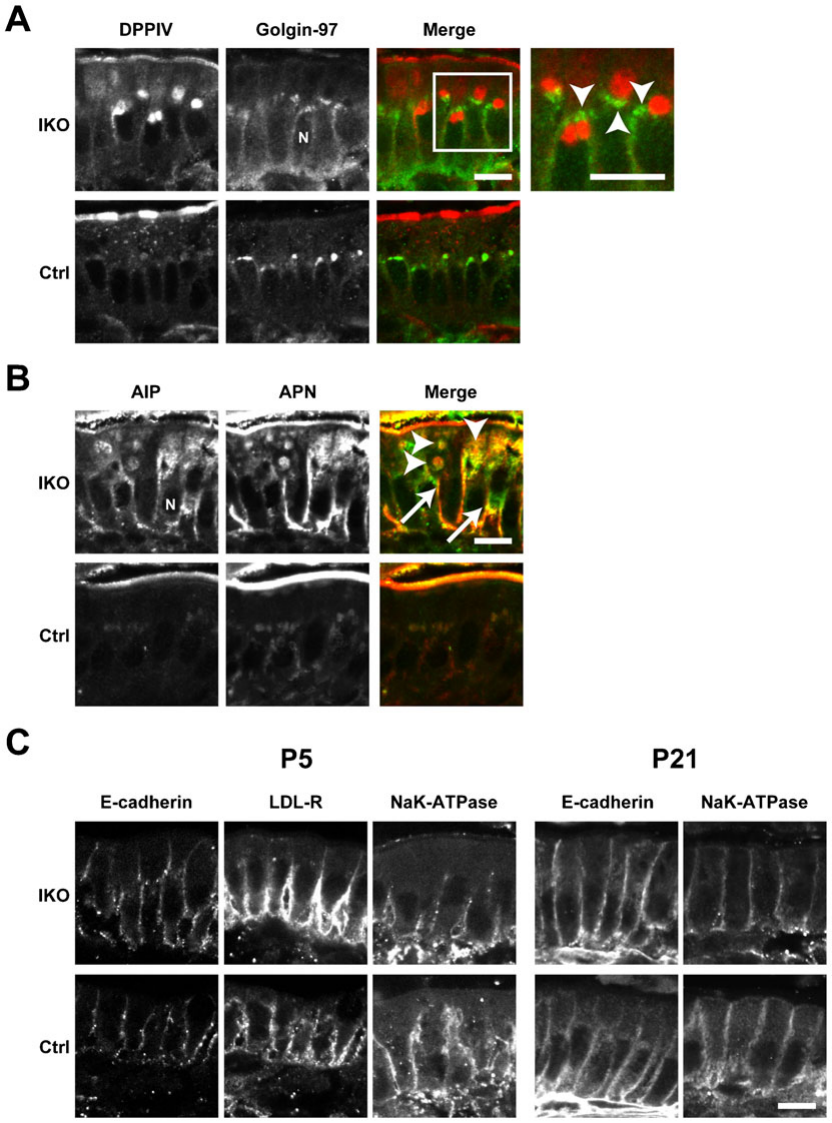
Localisation of apical and basolateral proteins in the small intestine of *Rab11a* IKO mice at P21. (A) DPPIV and the TGN marker Golgin-97 (arrowheads) do not colocalise in intestinal epithelial cells from IKO mice at P21. High-magnification insets (white boxes) are shown to the right of these panels. (B) Localisation of the apical proteins AlP and APN in control (Ctrl) and IKO cells at P21. Merged figures of AlP and APN are shown on the right. Intracellular vacuoles of AlP and APN are indicated by arrowheads. Basolateral localisation is indicated by arrows. (C) Localisation of the basolateral proteins E-cadherin, Na^+^K^+^-ATPase, and LDL-R in epithelial cells of the small intestine in Ctrl and IKO mice is not different at P5 and P21. Scale bars: 10 µm. N, nucleus.

To determine the role of Rab11a in the intestinal epithelial cells, we crossed the *Rab11a^flox/flox^* mice with *Villin-Cre* mice (Madison et al., 2002) (Fig. 1C). We confirmed the loss of Rab11a specifically in the intestine of intestine-specific *Rab11a* knockout (IKO) mice (*Villin-Cre; Rab11a^flox/flox^*) by Western blot analysis (Fig. 5A). The IKO mice were born with smaller body size than controls and most died within 2 weeks after birth (Fig. 1D,E and supplementary material Table S1). When we examined the distribution of apical and basolateral proteins in the IKO mice by immunofluorescence, we found mislocalisation of apical proteins in intestinal epithelial cells (Figs 2, 3). The apical protein dipeptidyl-peptidase 4 (DPPIV) distributed in the cytoplasm and colocalised with the lysosome marker lysosomal-associated membrane protein 2 (Lamp2) at P5 and P21 (Fig. 2, arrows). In addition, DPPIV was found to be mislocalised to the basolateral plasma membrane, which was evident in the epithelial cells at P5 (Fig. 2B, arrowheads). Accumulated DPPIV did not colocalise with the trans-Golgi network (TGN) marker Golgin97 (Fig. 3A). Other apical proteins, such as alkaline phosphatase (AlP) and aminopeptidase N (APN), were also found to accumulate within the cytoplasm (arrowheads) and mislocalised to the basolateral plasma membrane (arrows) in the IKO mice (Fig. 3B). In contrast, no basolateral proteins were mislocalised at P5 and P21 (Fig. 3C).

**Fig. 4.**
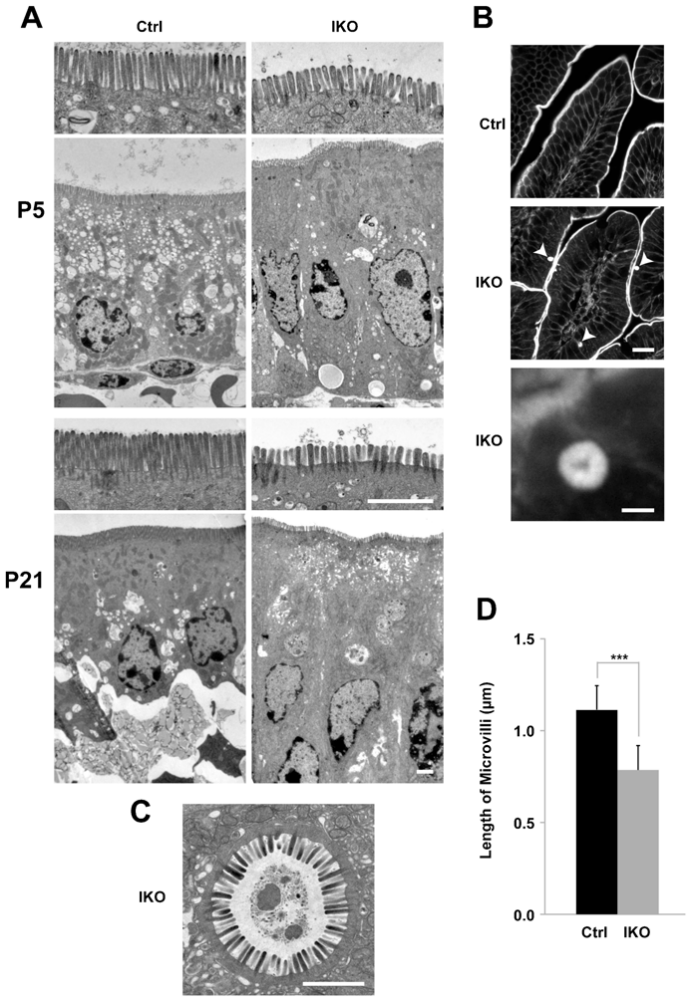
Microvillus atrophy and inclusions in *Rab11a* IKO mice. (A) Electron micrographs of P5 and P21 intestinal cells. Apical microvilli (first and third panels from top) are shorter in knockout cells (IKO) than in control (Ctrl) cells. (B) Sections of intestinal cells at P21 stained with phalloidin. F-actin localisation is prominent in the apical plasma membranes in villi of control intestines (top panel). Phalloidin-positive intracellular spheroids are evident in mutant villi (arrowheads in the middle panel). An enlarged view is shown in the bottom panel. (C) A microvillus inclusion body from a P21 IKO intestinal epithelial cell visualised using electron microscopy. (D) A graph showing microvillus length in epithelial cells at P5. Values represent mean ± s.d. (n>40 cells randomly selected from three mice per group; *** *P*<0.001; Student's *t*-test). Scale bars: 2 µm (A,C), 20 µm (B, middle), 2 µm (B, bottom).

The accumulation of apical proteins was previously observed in *Rab8a* knockout mice and *Rab8a/Rab8b* double-knockout mice (Sato et al., 2007; Sato et al., 2014). Since we also reported a shortening of microvilli and microvillus inclusion bodies in these mice, we observed these phenotypes in the small intestine of *Rab11a* IKO mice using electron and light microscopy (Fig. 4). Next, we analysed the terminal web, which is an actin-based filamentous structure associated with microvilli. Though the terminal web was stained by alpha-actinin (Geiger et al., 1979), there was no obvious difference in the width of this structure between the control and *Rab11a* IKO mouse (supplementary material Fig. S4A,B). This result indicates that the terminal web was not significantly affected in *Rab11a* IKO.

### Decreased apical protein levels in the small intestine of *Rab11a* IKO mice cause starvation

**Fig. 5.**
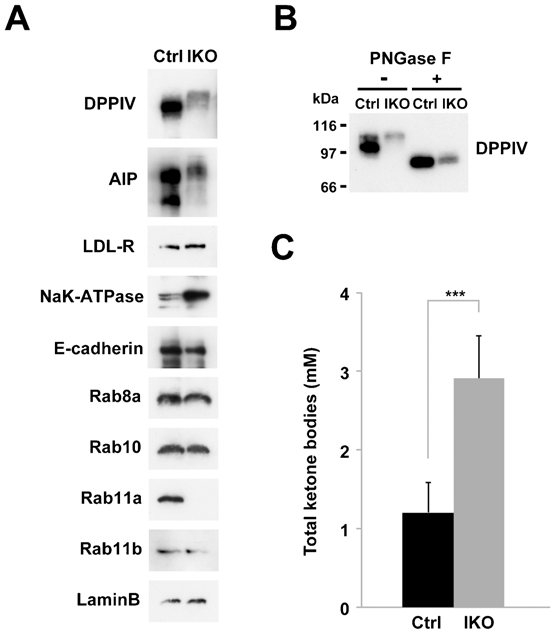
Quantification of a starvation marker and protein levels. (A) Western blot analysis of protein levels in crude extracts from the small intestines of control (Ctrl) and IKO mice at P5. Small intestine extracts were analysed by Western blot analysis using antibodies against various apical proteins (DPPIV and AlP), basolateral proteins (LDL-R, Na^+^K^+^ATPase, and E-cadherin), Rabs (Rab8a, 10, 11a, and 11b), and a loading control (Lamin B). (B) Treatment of peptide *N*-glycosidase F (PNGase F) in lysates from the small intestine of control (Ctrl) and IKO mice at P5. The samples were analysed by Western blotting using anti-DPPIV antibody. (C) Amounts of total ketone bodies in control (Ctrl) and IKO mice at P5. Values represent means ± s.d. from 4–10 mice. ***P<0.001; Student's *t*-test.

When we assessed the levels of apical and basolateral proteins in the small intestine by western blot, the levels of apical proteins (DPPIV and AlP) were lower in the IKO mice than those in the control mice. In addition, the molecular weights of DPPIV and AlP of the IKO mice appeared higher than those of the control mice (Fig. 5A). As we postulated that the apparent higher molecular weights were due to abnormal glycosylation, we treated DPPIV with peptide *N*-glycosidase (PNGase F) to remove *N*-linked carbohydrates. After PNGase F treatment, the molecular weight of DPPIV of IKO became similar to that of control (Fig. 5B), indicating there is no significant difference in the molecular weight of non-glycosylated protein between IKO and control. In contrast, the localisation of basolateral proteins appear intact (Fig. 3C). However, the amount of Na^+^K^+^-ATPase, one of basolateral proteins, was increased in the IKO than that in the control (Fig. 5A). However, the amount of other basolateral markers (LDL-R and E-cadherin) were not changed. Thus, the loss of Rab11a affects the amount of some basolateral protein (Na^+^K^+^-ATPase). As the Rab11a IKO mice were runted and died at early postnatal period like *Rab8a* KO mice (Sato et al., 2007), we examined whether IKO mice were also starved to death. We observed an increase in the level of a starvation marker (total ketone bodies) in the blood of the IKO mice (Fig. 5C). These data suggest that similar molecular mechanisms are responsible for lethality in the *Rab11a* IKO mice and *Rab8a* KO mice (Sato et al., 2007). Namely, the mislocalisation of apical proteins (e.g., enzymes and transporters) in these knockout mice may lead to decreased nutrient uptake, causing starvation and death.

### Rab11a functionally interacts with Rab8a and myosin Vb *in vivo*

**Fig. 6.**
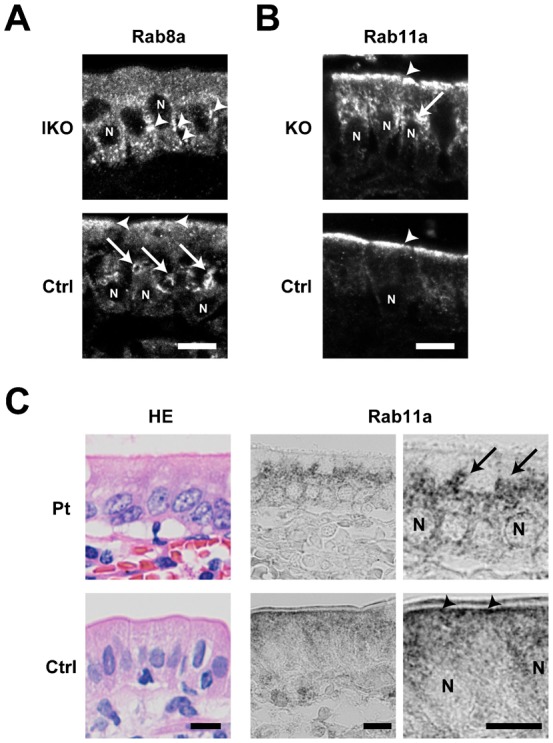
Spatial interdependence between Rab11a, Rab8a, and myosin Vb. (A) Localisation of Rab8a in epithelial cells of the small intestine in control (Ctrl) and *Rab11a* IKO mice at P5. Rab8a is mainly localised on perinuclear punctate structures (arrowheads) below the nucleus (N) in IKO cells, whereas it is localised on large vacuolar structures (arrows) above the nucleus in control cells. (B) Localisation of Rab11a in epithelial cells of the small intestine in Ctrl and *Rab8a* KO mice at P7. Rab11a is exclusively localised below the apical plasma membrane (an arrowhead) in Ctrl epithelial cells, but it is also localised punctate structures (an arrow) above the nucleus in *Rab8a* KO epithelial cells. (C) Localisation of Rab11a in epithelial cells of the small intestine from a control (Ctrl) and a microvillus atrophy patient (Pt). Rab11a is localised below the apical plasma membrane in control intestinal cells (arrowheads), but it is localised perinuclear structures in patient's intestinal cells (arrows). HE Scale bars: 10 µm. N, nucleus.

As the phenotypes of *Rab11a* IKO mice were similar to those of *Rab8a* knockout mice, we thought that Rab11a and Rab8a might function in cooperation for apical transport pathway to some extent. We then examined the localisation of Rab8a in the epithelial cells of the IKO mice and *vice versa*. Indeed, we found that Rab8a was mislocalised in the IKO mice and that Rab11a was mislocalised in *Rab8a* knockout mice compared with control mice, demonstrating a functional relationship between these proteins (Fig. 6A,B). Both Rab8a and Rab11a are known to bind myosin Vb (Lapierre et al., 2001; Roland et al., 2007; Jin et al., 2011). Therefore, to determine whether myosin Vb protein is involved in the localisation of Rab11a, we compared small intestine samples from a microvillus atrophy patient and a healthy individual (Fig. 6C), as microvillus atrophy patients are known to have mutations in the *myosin Vb* gene (Müller et al., 2008). In the healthy small intestine, Rab11a localised subapically (Fig. 6C). However, in the small intestine of the microvillus atrophy patient, Rab11a localised closer to the nucleus (Fig. 6C). Taken together, these results indicate that Rab11a appears to functionally interact with Rab8a and myosin Vb *in vivo*.

### Rab11a is important for apical transport in the intestine during early postnatal period

**Fig. 7.**
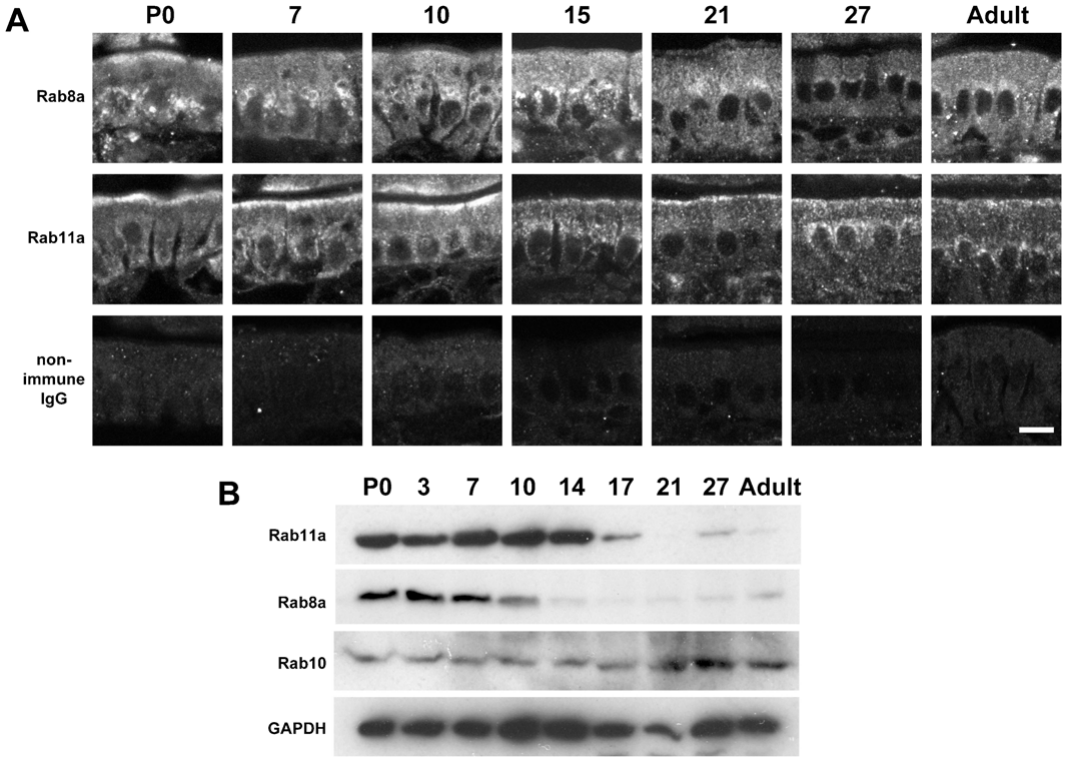
Localisation of Rab8a and Rab11a and quantification of Rab11a, Rab8a, and Rab10 in the small intestine during postnatal development. (A) Localisation of Rab8a (top), Rab11a (middle) and non-immune rabbit IgG (bottom), as determined by immunofluorescence, during postnatal (P) days in wild-type epithelial cells of the small intestine. (B) Levels of Rab11a, Rab8a, and Rab10 in the wild-type small intestine during postnatal development. Scale bar: 10 µm.

Although the phenotypes of *Rab11a* IKO mice and *Rab8a* knockout mice were similar, the onset of the phenotype was earlier in the IKO mice (begin dying from 5 days after birth) than in the *Rab8a* knockout mice (begin dying from 3 weeks after birth) or *Rab8a/8b* double-knockout mice (begin dying from 2 weeks after birth). To investigate these differences, we performed Western blot and immunofluorescence analyses on wild-type small intestines during postnatal development. As determined by immunofluorescence, Rab11a primarily localises to the subapical region from birth until 10 days after birth (Fig. 7A). In contrast, Rab8a primarily localises to the perinuclear region and is nearly absent by P21, as previously described (Sato et al., 2007) (Fig. 7A). These data suggests that Rab11a and Rab8a might function during different steps of the apical transport process in the epithelial cells. As determined by Western blot analysis, Rab8a protein levels increased over the first 10 days after birth and then gradually faded, as previously described (Sato et al., 2007) (Fig. 7B). In contrast, Rab10 was consistently expressed throughout postnatal development, although the levels moderately increased 3 weeks after birth (Fig. 7B). Rab11a was abundantly expressed until 2 weeks after birth (Fig. 7B). These data demonstrate that the IKO mice die during the period when Rab11a levels are normally most abundant, suggesting the importance of this protein in apical transport in the small intestine during the early postnatal period.

## DISCUSSION

In this study, we generated conventional (systemic), brain-specific, and intestine-specific *Rab11a* knockout mice. The conventional knockout mice were embryonic lethal (supplementary material Table S1). The brain-specific knockout mice displayed no overt phenotypes (supplementary material Figs S1–S3). However, we found that Rab11a is essential for the proper localisation of apical proteins in the intestine (Figs 2, 3). Rab11 is known to localise to apical recycling endosomes in polarised cells (Casanova et al., 1999), and it has been reported that Rab11 is involved in shuttling membranes from intracellular vesicles to the plasma membrane in a variety of organisms (yeast, fly, worm, and mouse) (Benli et al., 1996; Li et al., 2007; Sato et al., 2008; Duman et al., 1999; Khandelwal et al., 2008; Khandelwal et al., 2013). Recently, it was shown that recycling endosomes are essential for biosynthetic transport from the TGN to plasma membrane by functionally disrupting recycling endosomes (Ang et al., 2004). Cresawn et al. also showed that apical recycling endosomes are an essential route for the biosynthetic transport of ‘raft’-associated apical cargos to the apical plasma membrane from the TGN (Cresawn et al., 2007). These reports suggest the involvement of Rab11 in the apical transport of several proteins through a number of biosynthetic pathways. Here, we showed that the loss of Rab11 leads to the mislocalisation of apical proteins in the small intestine using *Rab11a* IKO mice (Figs 2, 3). Furthermore, as we detected abnormal *N*-glycosylation of apical proteins in *Rab11a* IKO mice (Fig. 5A,B), Rab11a is likely to be involved in apical biosynthetic transport in small intestinal epithelial cells. We presume that depletion of Rab11a decelerates apical protein transport through the Golgi apparatus and increases *N*-glycosylation of apical proteins. We should perform more experiments in future to test this idea.

Rab11 proteins have a number of binding partners, including myosin Vb (Lapierre et al., 2001; Hales et al., 2001), which is also known to bind Rab8 and Rab10 (Roland et al., 2007; Roland et al., 2009). The human *myosin Vb* gene is mutated in microvillus inclusion disease, which is characterised by the mislocalisation of apical proteins, shortening of microvilli and microvillus inclusion bodies. These phenotypes are also observed in *Rab8a* knockout mice and *Rab8a/Rab8b* double-knockout mice (Sato et al., 2007; Sato et al., 2014; Müller et al., 2008), suggesting a close functional relationship between myosin Vb and Rab8. Here, we observed shorter microvilli and microvillus inclusion bodies in *Rab11a* IKO mice (Fig. 4) and showed that Rab11a and Rab8a depend on one another for proper localisation (Fig. 6A,B). We also observed that the localisation of Rab11a in the small intestinal tissue from a microvillus atrophy patient was different from that in a healthy intestinal tissue (Fig. 6C). These results indicated that the localisation of Rab11a, Rab8a, and Myosin Vb were dependent on one another, suggesting the functional relationship between these proteins. Notably, the localisation of Rab11a was obviously different from that of Rab8a (Fig. 7A). It is likely that Rab11a and Rab8a regulate different steps of the same apical transport pathway by utilizing the common binding protein, Myosin Vb. This is similar to the case in yeast. Myo2p which is a homolog of mammalian Myosin Vb sequentially associates with Ypt31p and Sec4p, homologs of Rab11a and Rab8a respectively (Santiago-Tirado et al., 2011; Mizuno-Yamasaki et al., 2010).

*Rab11a* IKO mice displayed a similar phenotype, such as mislocalisation of apical proteins, shortening of microvilli, and microvillus inclusion body formation, to those of *Rab8a* knockout and *Rab8a/Rab8b* double-knockout mice (Figs 2–4). However, the phenotype in the IKO mice was apparently much earlier than in *Rab8a/Rab8b* double-knockout mice. Therefore, Rab11a appears to be a key molecule in apical transport, even when Rab8a plays minor roles for it. Furthermore, at a later postnatal period (P21) when Rab8a is required, *Rab11a* IKO mice show a very similar phenotype to Rab8a KO mice. Based on these results, we conclude that Rab11a is essential for apical transport independently of Rab8, and indeed, Rab11a appears to play an even more critical role in this process than Rab8 (Fig. 7).

Finally, *Rab11a* BKO mice appeared normal and displayed no obvious abnormalities (supplementary material Figs S1–S3). Previous studies have reported that Rab11 is essential for neurite elongation through a number of effectors (Shirane and Nakayama, 2006; Takano et al., 2012). However, compensation by other Rab11 family proteins, particularly Rab11b, cannot be ruled out, as mice deficient for another Rab11 family protein, Rab25, displayed no overt neuronal phenotype. Regardless, we found this result surprising due to the variety of localisations and roles proposed for each Rab11 family (Lapierre et al., 2003). To more fully characterise the role of Rab11 family members in the nervous system, it will be necessary to examine brain-specific *Rab11a* knockout mice in greater detail, as well as to generate *Rab11b* knockout mice and/or brain-specific *Rab11a*, *Rab11b*, and* Rab25* double- or triple-knockout mice.

## MATERIALS AND METHODS

### Generation of *Rab11a* knockout mice

All animal procedures were performed according to the guidelines of the Animal Care and Experimentation Committee of Osaka University, and all animals were bred at the Institute of Animal Experimental Research of Osaka University.

*Rab11a* knockout mice were generated largely as described previously (Harada et al., 1994). *Rab11a* knockout ES clones (*Rab11a^tm1a(KOMP)Wtsi^*) were provided by the UC Davis KOMP Repository.

Targeted clones were confirmed by Southern blotting analysis (Fig. 1B) prior to blastocyst injection. To generate *Rab11a ^flox/+^*mice, we crossed *Rab11a^neo/+^* mice with actin-flippase transgenic mice (B6; SJL-Tg (ACTFLPe) 9250 Dym/J) (Jackson Laboratory, Bar Harbor, ME, USA). To generate brain-specific and intestine-specific *Rab11a*-knockout mice, we crossed *Rab11a^flox/+^* mice with *Nestin-Cre* transgenic mice (Tronche et al., 1999) and *Villin-Cre* transgenic mice (Madison et al., 2002) (Jackson Laboratory, Bar Harbor, ME, USA), respectively.

Genotypes in animals were confirmed by PCR using genomic DNA from tails of control and knockout mice. Primers used for genotyping PCR were as follows: primer ‘F’, 5′-TAA GCC TCG TGC CTC CTG TTT TAA-3′; primer ‘R1’, 5′-ACC TTG GGC AAG AAC ATA AAG TGA-3′; primer ‘R2’, 5′-ATT CCT ATT TAG GAA CTC ACA CCC-3′; primers ‘Cre’, 5′-AGG TTC GTT CTC TCA TGG A-3′ (*cre* forward); 5′-TCG ACC AGT TTA GTT ACC C-3′ (*cre* reverse).

### Immunofluorescence and immunoblotting

Mice at different postnatal days were anesthetised and fixed by intracardial perfusion or immersion with 3% paraformaldehyde in 0.1 M phosphate buffer (pH 7.2) and further fixed for 2 h in the same fixative. For cryoprotection, the fixed tissues were soaked in a series of 4, 10, 15, and 20% sucrose in 0.1 M phosphate buffer (pH 7.2) at 4°C for at least 30 min for each step. The tissues were then frozen in isopentane chilled in liquid nitrogen and stored in liquid nitrogen until cryosectioning. The tissues were cut into 5–10 µm sections. Western blotting (WB) was performed as previously described (Sato et al., 2011) with 10 µg of protein per lane.

The following antibodies and dyes were used in this study (dilutions refer to immunofluorescence (IF) assays, unless otherwise noted): actinin-4 (1:100; Alexis biochemicals, San Diego, CA, USA); AlP (1:1000 for WB, 1:100 for IF; Rockland, Gilbertsville, PA, USA); APN (1:100; BMA Biomedicals, Augst, Switzerland); Arl13b (1:1000; ab136648; Abcam, Cambridge, MA, USA); calbindin (1:100; Santa Cruz Biotechnology, Dallas, TX, USA); Cux1 (1:50; Santa Cruz Biotechnology, Dallas, TX, USA); dipeptidyl-peptidase 4 (DPPIV; 1:100; R&D Systems, Minneapolis, MN, USA); 4′,6-Diamidine-2′-phenylindole dihydrochloride (DAPI; 1:1000; Roche, Basel, Switzerland); E-cadherin (1:1000 for WB; BD, San Jose, CA, USA, 1:50 for IF; TAKARA, Shiga, Japan); GAPDH (1:2000 for WB; EMD Millipore, San Diego, CA, USA); Golgin-97 (1:100; Yoshimura et al., 2004); Lamp2 (1:100; clone Abl-93; Developmental Studies Hybridoma Bank, Iowa, USA); Lamin B (1:500 for WB; Santa Cruz Biotechnology, Dallas, TX, USA); low density lipoprotein receptor (LDL-R; 1:1000 for WB, 1:100 for IF, R&D Systems, Minneapolis, MN, USA); microtubule-associated protein 1A (MAP1A; 1:100; Shiomura and Hirokawa, 1987); Na^+^-K^+^ ATPase (1:1000 for WB; clone C464.6; Upstate Biotechnology, 1:100 for IF; Homareda et al., 1993); Phalloidin–Tetramethylrhodamine B isothiocyanate (1:1000; Sigma-Aldrich, St. Louis, MO, USA); Rab8a (1:1000 for WB, 1:100 for IF; Sato et al., 2007); Rab10 (1:500 for WB; Cell Signaling Technology, Danvers, MA, USA); Rab11b (1:500 for WB; Aviva Systems Biology, San Diego, CA, USA); synaptophysin (1:1000; EMD Millipore, Billerica, MA, USA), and Tbr1 (1:500; EMD Millipore, Billerica, MA, USA). Alexa 488- or Alexa 594-labelled species-specific secondary antibodies (1:400; Life Technologies, Carlsbad, CA, USA) were used.

The rabbit polyclonal antibody against Rab11a was raised using a bacterially expressed GST-fused Rab11a protein fragment (C-terminal 30 amino acids) encoded by pGEX4T1. The antisera were affinity purified prior to use in the experiments (1:500 for WB and 1:100 for IF).

The stained sections were analysed by confocal microscopy (FV1000D, Olympus, Tokyo, Japan or LSM510 META, Carl Zeiss Japan, Tokyo, Japan), as previously described (Sato et al., 2007).

### Nissl staining

Paraformaldehyde-fixed brains were sectioned in 16 µm thick with cryostat (Leica, Germany). The sections were washed with water and then stained with Toluidine blue O (Waldeck GmbH&Co. KG, Division Chroma, Muenster, Germany) solution (0.01% Toluidine blue O, 0.06 M sodium citrate and 0.08 M Na_2_HPO_4_) for 15 min at room temperature. After washing in water, the sections were analysed by using a BX61 light microscope (Olympus Corporation, Tokyo, Japan).

### Peptide *N*-glycosidase F (PNGase F) treatment

Small intestinal tissue derived from control or IKO mice was lysed by the buffer containing 10 mM Tris-HCl, pH 7.8, 0.5 M NaCl, 1 mM ethylenediaminetetraacetic acid, 1% Nonidet P-40) with protease inhibitor cocktail (Roche, Basel, Switzerland). After centrifugation (15,000 g) for 10 min at 4°C, the supernatant was boiled in 0.1 M 2-mercaptoethanol and 0.5% SDS for 10 min. After boiling, the 50 µg of protein was incubated for 16 h at 37°C with 100 mM Tris-HCl (pH 8.6), 1% Nonidet P-40 and 40 mU/ml PNGase F (TAKARA, Shiga, Japan). The sample was subjected to SDS-PAGE and analysed by Western blotting.

### Measurement of a starvation marker

Approximately 100 µl of the blood was sent to SRL Inc. (Tokyo, Japan) and analysed the measure of total ketone bodies (acetoacetic acid and 3-hydroxy-butyrate).

### Electron microscopy

Mice were perfused or immersed with 2% paraformaldehyde and 2.5% glutaraldehyde in 0.1 M cacodylate buffer (pH 7.4). The tissues were then dissected, fixed for another 2 h at RT, and treated with 1% OsO_4_ in 0.1 M cacodylate buffer followed by 0.5% uranyl acetate in water. The samples were dehydrated and embedded in Epon, and the thin sections were post-stained with uranyl acetate and lead citrate, as previously described (Harada et al., 1990; Harada et al., 1994). The sections were then examined by electron microscopy (H7650; Hitachi, Tokyo, Japan) at 80 kV.

### Image processing and quantification

Images were processed using Adobe Photoshop® (Adobe Systems, Inc., CA, USA) version 7.0.

### Analysis of human samples by immunohistochemistry

We used small intestine samples from an early-onset microvillus inclusion disease patient. The male patient was diagnosed with microvillus inclusion disease by electron-microscopic examination of the jejunal epithelial cells at 4 months of age. Detailed descriptions of this patient are available in previous papers (Kagitani et al., 1998). This study was approved by the Ethics Committee of Osaka University School of Medicine based on the written, informed consent of each subject. The small intestine samples were obtained following small intestine transplantations in the patients, and they were fixed in formaldehyde and embedded in paraffin.

The paraffin-embedded sections of diseased and control human small intestine tissue were prepared on license from the ethics committee of Osaka university hospital. The paraffin slides were rehydrated in a descending ethanol series following deparaffinisation with Clear Plus. After rinsing with 1× phosphate-buffered saline (PBS; pH 7.4), the slides were treated with blocking solution (PBS containing 3% bovine serum albumin (Sigma) and 5% normal goat serum (Gibco)) for 1 hour at room temperature (RT). The slides were then incubated overnight at 4°C with a polyclonal rabbit anti-Rab11a antibody (1:100) and a polyclonal rabbit anti-alkaline phosphatase antibody (1:100; Rockland Immunochemicals Inc., PA, USA) in blocking solution. The slides were washed with PBS and then treated with a biotin-conjugated goat anti-rabbit IgG secondary antibody (1:200; Vector Laboratories Inc., CA, USA) for 30 min at RT in PBS. Subsequently, the slides were incubated in 1.5% H_2_O_2_ for 30 min at RT to eliminate endogenous peroxidases. Following amplification with the avidin-biotin complex (ABC kit; Vector Laboratories), visualisation of the reaction products was carried out with 50 mM Tris-buffered saline (TBS; pH 7.4) containing 1.25% DAB and 0.75% hydrogen peroxide. The slides were immersed in 50 mM TBS to stop the reaction, followed by hematoxylin treatment as a counter-stain. Finally, following dehydration, the slides were coverslipped and sealed with Entellan (Merck, Darmstadt, Germany). Stained samples of non-treated small intestines were also prepared as controls. Control samples were examined using normal rabbit IgG (1:100; Dako, Glostrup, Denmark) instead of the primary antibody during the incubation process. We performed hematoxylin-eosin (HE) staining using standard histological procedures. All slides were analysed using a BX61 light microscope (Olympus Corporation, Tokyo, Japan).

## Supplementary Material

Supplementary Material
