# Supplementary material for: Rab11a is required for apical protein localisation in the intestine
Source: Biol Open. 2014 Dec 19;4(1):86–94. doi: 10.1242/bio.20148532 (PMC4295169; doi:10.1242/bio.20148532)
Supplement: Supplementary Material [file supp_4_1_86__index.html]

Rab11a is required for apical protein localisation in the intestine — Rab11a is required for apical protein localisation in the intestine — Supplementary Material 

# Rab11a is required for apical protein localisation in the intestine

## bio.20148532 Supplementary Material

**Files in this Data Supplement:**

- Supplementary Material - Tomoaki Sobajima et al. doi: 10.1242/bio.20148532
