# Supplementary material for: Rab11a is required for apical protein localisation in the intestine
Source: Biol Open. 2014 Dec 19;4(1):86–94. doi: 10.1242/bio.20148532 (PMC4295169; doi:10.1242/bio.20148532)
Supplement: Supplementary Material [file supp_bio.20148532_bio.20148532-s1.pdf]

Biology Open (2015) 000, 1–9 doi:10.1242/bio.20148532

Supplementary Material

Tomoaki Sobajima et al. doi: 10.1242/bio.20148532

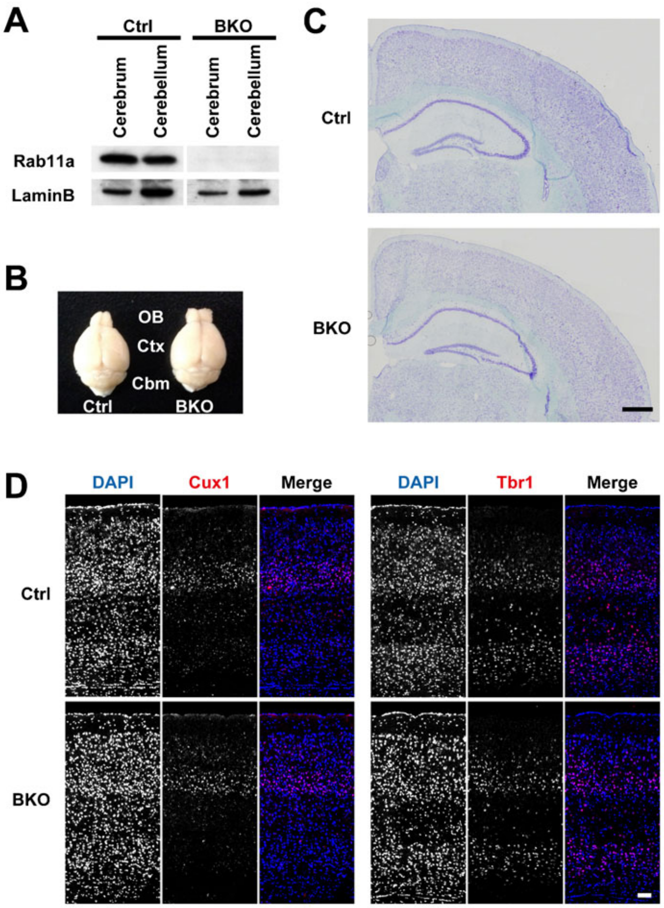

Fig. S1. Deletion of *Rab11a* with *Nestin-Cre* has no obvious effects on brain development. (A) Western blotting for Rab11a and Lamin B in lysates from the cerebrum and cerebellum. (B) Adult brains of control (Ctrl) and *Rab11a* BKO mice. (C) Nissl staining of coronal brain sections from Ctrl and BKO mice. (D) Localisation of Cux1 and Tbr1 (red) with DAPI (blue) in the dorsolateral region of the cerebral cortex. Scale bars: 500 µm (C), 100 µm (D). OB, olfactory bulb; Ctx, cerebral cortex; Cbm, cerebellum.

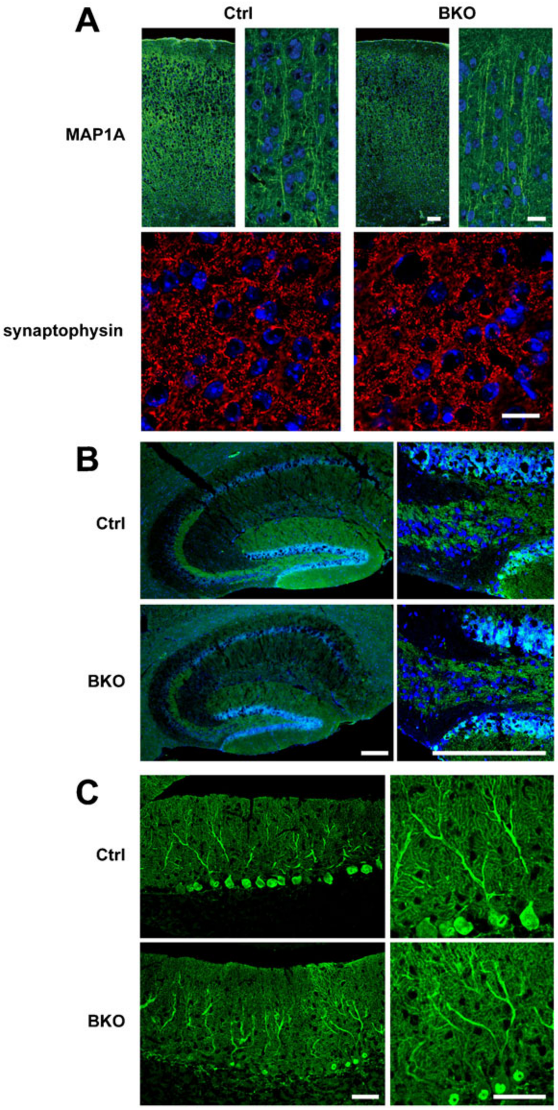

Fig. S2. Deletion of *Rab11a* with *Nestin-Cre* has no obvious effects on axon and dendrite formation. (A) Localisation of MAP1A (green) and synaptophysin (red) with DAPI (blue) in the dorsolateral region of the cerebral cortex. (B) Localisation of calbindin (green) with DAPI (blue) in dentate granule cells of the hippocampus. (C) Localisation of calbindin (green) in Purkinje cells of the cerebellum of control (Ctrl) and BKO mice. Scale bars: 100 µm, inset 20 µm (A, upper panels), 20 µm (A, lower panels), 200 µm (B), 50 µm (C).

Biology Open

 Biology Open (2015) 000, 1–9 doi:10.1242/bio.20148532

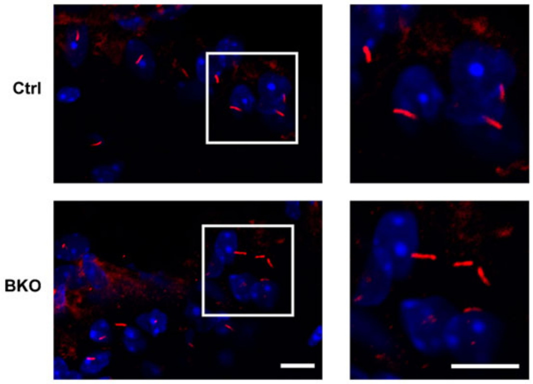

Fig. S3. Deletion of *Rab11a* with *Nestin-Cre* has no obvious effects on ciliogenesis. Localisation of Arl13b (red) with DAPI (blue) in the paraventricular hypothalamus region of control (Ctrl) and BKO mice. Scale bars: 10 μm.

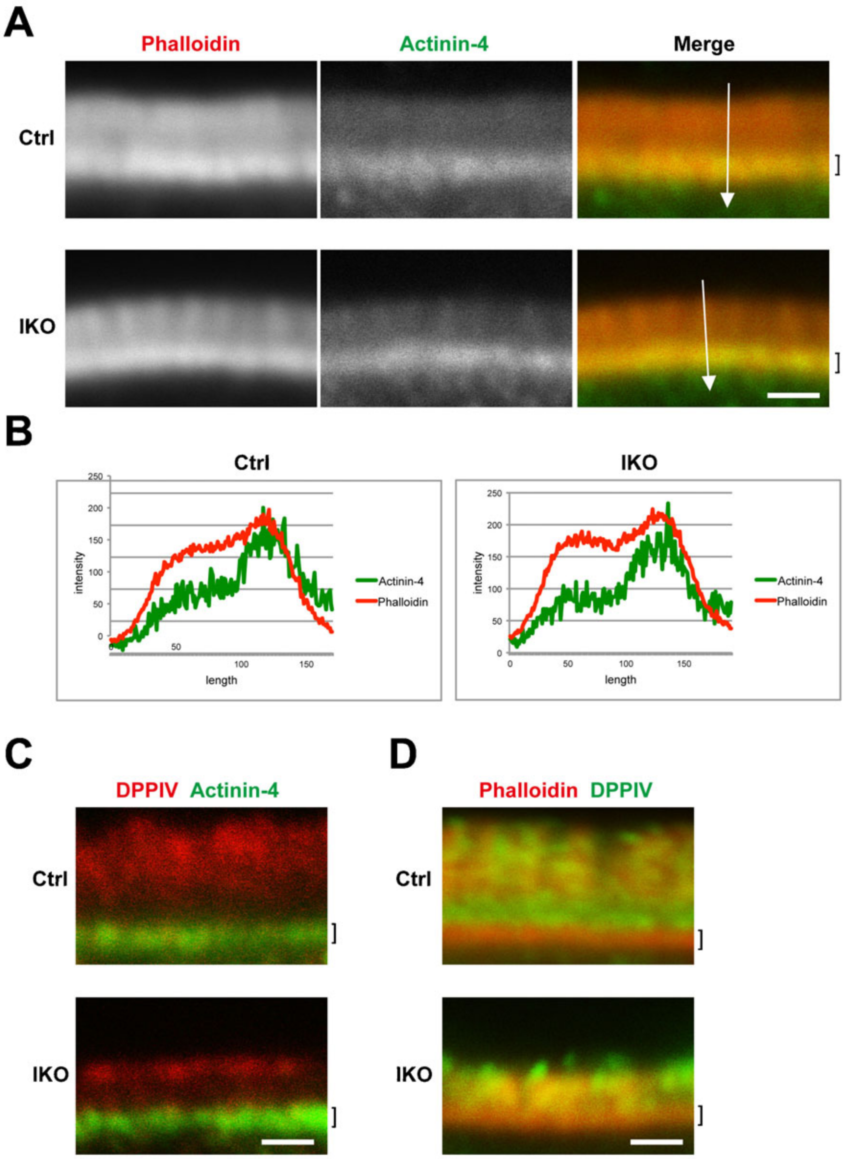

Fig. S4. Deletion of *Rab11a* with *Villin-Cre* has no obvious effects on terminal web formation. (A) Localisation of F-actin (phalloidin: red) and actin binding protein (actinin-4: green) (B) Intensity plots of phalloidin (red lines) and actinin-4 (green lines) in control (left) and IKO (right) apical regions marked by white arrows in (A). (C) Localisation of an apical membrane protein (DPPIV: red) and actinin-4 (green). (D) Localisation of phalloidin (red) and DPPIV (green). Actinin-4 is colocalised with phalloidin (A) but not with DPPIV (C), which indicates the actinin-4 positive region is terminal web (right bracket marks in A, C and D). Scale bars: 1 μm.

Biology Open

Biology Open (2015) 000, 1–9 doi:10.1242/bio.20148532

Table S1. Genotype distribution of offspring

Rab11a$^{neo/+}$×*Rab11a*$^{neo/+}$

	Rab11a$^{+/+}$	*Rab11a*$^{neo/+}$	*Rab11a*$^{neo/neo}$	Total
Adult	19	45	0	64
E14.5	5	6	0	11
E11.5	3	9	0	12

Nestin-Cre; Rab11a$^{flox/+}$×*Rab11a*$^{flox/flox}$

	Rab11a$^{flox/+}$	*Rab11a*$^{flox/flox}$	*NC; Rab11a*$^{flox/+}$	*NC; Rab11a*$^{flox/flox}$	Total
Observed	11	6	10	8	35
Observed (%)	31	17	29	23	100
Expected (%)	25	25	25	25	100

Villin-Cre; Rab11a$^{flox/+}$×*Rab11a*$^{flox/flox}$

	Rab11a$^{flox/+}$	*Rab11a*$^{flox/flox}$	*VC; Rab11a*$^{flox/+}$	*VC; Rab11a*$^{flox/flox}$	Total
Observed	26	24	21	19	90
Observed (%)	29	27	23	21	100
Expected (%)	25	25	25	25	100

Mating of the following genotypes: heterozygotes (*Rab11a*$^{neo/+}$)×heterozygotes (*Rab11a*$^{neo/+}$), *Nestin-Cre*; *Rab11a*$^{flox/+}$×*Rab11a*$^{flox/flox}$, and *Villin-Cre*; *Rab11a*$^{flox/+}$×*Rab11a*$^{flox/flox}$. *NC*, *Nestin-Cre*; *VC*, *Villin-Cre.*
